# Analysis of the relationship between the mutation site of the SLC39A4 gene and acrodermatitis enteropathica by reporting a rare Chinese twin: a case report and review of the literature

**DOI:** 10.1186/s12887-020-1942-4

**Published:** 2020-01-27

**Authors:** Wei Zhong, Chao Yang, Lei Zhu, Yu-Qi Huang, Yong-Feng Chen

**Affiliations:** 1 Guangdong Medical College, College of Dermatology, Anhui Medical University, Guangzhou, China; 2 Guangdong Provincial Dermatology Hospital, Guangzhou, China; 3 Dermatology Hospital of Southern Medical University, Guangzhou, China

**Keywords:** Acrodermatitis enteropathica, SLC39A4 gene, Mutation, Genotype-phenotype

## Abstract

**Background:**

Acrodermatitis enteropathica (AE) is a rare autosomal recessive hereditary skin disease caused by mutations in the SLC39A4 gene and is characterized by periorificial dermatitis, alopecia and diarrhoea due to insufficient zinc absorption. Only one of the three known sets of twins with AE has genetic information. This case reports the discovery of new mutation sites in rare twin patients and draws some interesting conclusions by analysing the relationship between genetic information and clinical manifestations.

**Case presentation:**

Here, we report a pair of 16-month-old twin boys with AE exhibiting periorificial and acral erythema, scales and blisters, while subsequent laboratory examination showed normal plasma zinc and alkaline phosphatase levels. Further Sanger sequencing demonstrated that the patients were compound heterozygous for two unreported *SLC39A4* mutations: a missense mutation in exon 5 (c.926G > T), which led to a substitution of the 309th amino acid residue cysteine with phenylalanine, a splice site mutation occurring in the consensus donor site of intron 5 (c.976 + 2 T > A). A family study revealed that the boys’ parents were heterozygous carriers of these two mutations.

**Conclusion:**

We identified a new compound heterozygous mutation in Chinese twins with AE, which consisted of two previous unreported variants in exon 5 and intron 5 of SLC39A4. We propose an up-to-date review that different mutations in SLC39A4 may exhibit different AE manifestations. In conjunction with future research, our work may shed light on genotype-phenotype correlations in AE patients and provide knowledge for genetic counselling and treatment for AE patients.

## Background

Acrodermatitis enteropathica (AE; OMIM 201100) is an autosomal recessive genetic disease that causes severe zinc deficiency and has an incidence rate of 1/500,000 [1]. The zinc deficiency is due to the SLC39A4 gene mutation, which limits the zinc absorption of the ZIP4 transporter in the small intestines, resulting in insufficient zinc absorption in the duodenum and jejunum [2]. Zinc is an essential coenzyme in metalloenzymes, including alkaline phosphatase; this enzyme regulates gene expression and is an important structural component of gene regulatory proteins, such as those required for intracellular binding of tyrosine kinases to T cell receptors [3]. Zinc can promote growth, sexual organ development and wound healing and has repairing effects on oral mucosa, hair, nails and other body parts [4]. Zinc deficiency can present in various clinical symptoms, such as growth retardation, decreased immune function and different skin or gastrointestinal injuries [5]. AE is classified as either hereditary or acquired and is characterized by periorificial dermatitis, alopecia and diarrhoea. These three symptoms simultaneously occur in only 20% of patients [6], usually during the weaning period of children, and they vary with age. Advanced AE symptoms may include neuropsychiatric disorders, hypogonadism, growth retardation and immune system dysfunction. Untreated patients with AE may eventually lead to multiple organ failure and death. Zinc deficiency accounts for 4% of morbidity and mortality in children aged from 6 months to 5 years worldwide [7]. Laboratory diagnosis requires the detection of zinc in serum, urine or hair, but the results are often unspecific and nonsensitive; thus, some patients with zinc levels can appear normal [2]. The zinc absorption test is cumbersome to use, and the SLC39A4 gene test can confirm the disease. Most clinicians rely on a zinc supplementation regimen (1~5 mg/kg) to predict clinical diagnosis with treatment outcomes [8]. The aforementioned regimen should be taken orally for a long time. If oral absorption is difficult, then it can be injected [6]. General treatment includes protein and vitamin supplementation and blood transfusion if necessary. Skin cleansing should be properly conducted to prevent infection.

## Case presentation

**Fig. 1:**
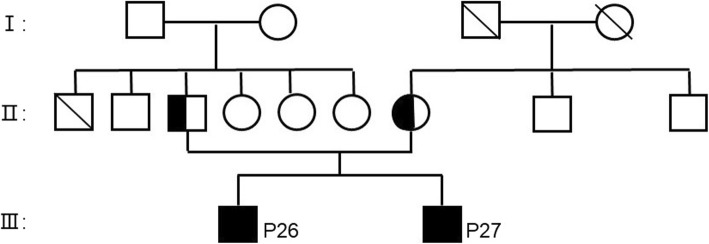
Pedigrees of the families. Icons corresponding to the individuals we examined are marked. People exhibiting a mutation in both alleles of SLC39A4 are identified by symbols filled in black. Heterozygote carriers for a unique mutation are symbolized by half-filled icons. By default, all the untested people, including some parents, are represented by white icons

We present the case of 16-month-old twin boys admitted to the hospital due to skin lesions that appeared 12 months after birth. The erythema of the oral region of the twins appeared one after the other, and a large area of erythema with a clear boundary and peeling was observed at the centre of the perianal portion. They came to our hospital due to ineffective treatment at the local hospital. The twins were born full term and breastfed for 7 months before weaning. In addition, the parents have a non-consanguineous marriage (Fig. 1).

**Fig. 2:**
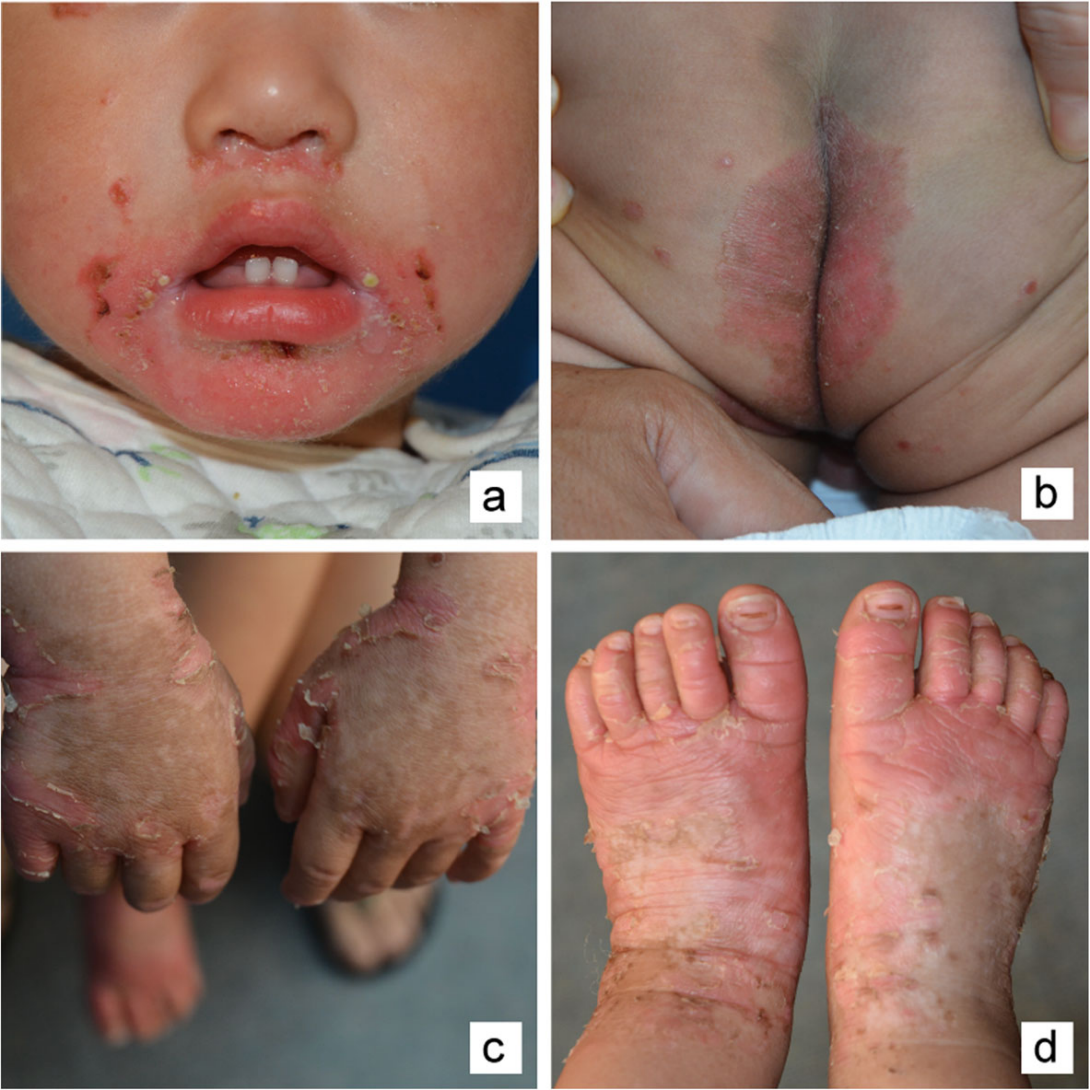
Clinical photo of one of the twin patients: **a** Mouth-based face. **b** The perianal area has erythema with yellow-black sputum. **c d** The blisters are visible on both hands and feet

**Fig. 3:**
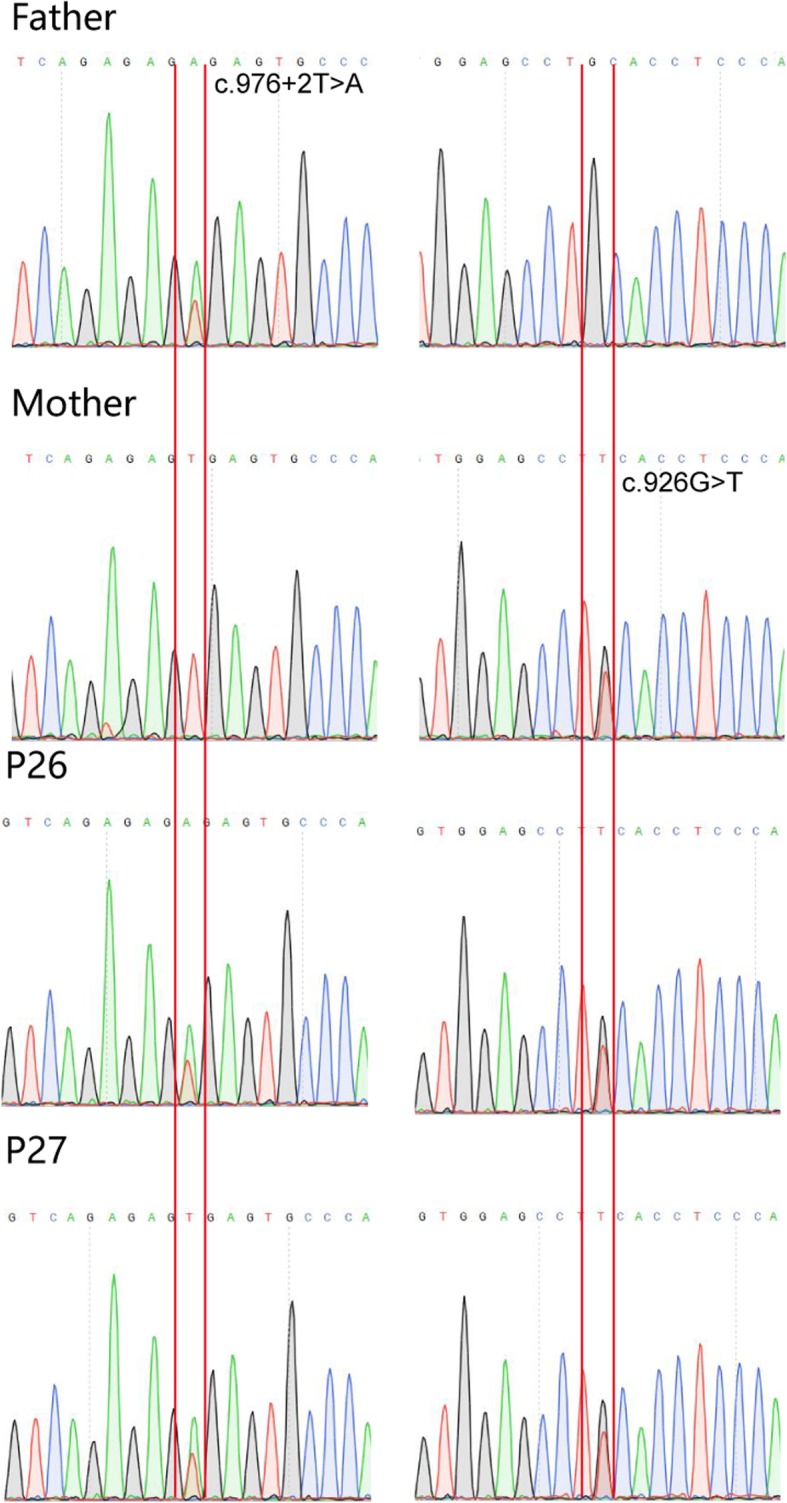
Identification of heterozygous mutations in the SLC39A4 gene, one from the twins’ father (c.976 + 2 T > A) and the other from their mother (c.926G > T)

The twins had normal physique, and no developmental delay was observed (older brother 9 kg, 78 cm; younger brother 10 kg, 79 cm). The skin was dry and dark, the mental state was poor, and the crying was weak. Alopecia was not observed. The patients presented poor spirit and intermittent manic episodes. Symmetrical erythema appeared on the perioral region, hands, wrists, knees, feet and genital and perianal areas (Fig. 2). Complete blood cell count, liver and kidney function tests, serum zinc (4.2 mg/L; reference range 3.7–7.3 mg/L) and alkaline phosphatase levels (45 U/L, reference range 37–147 U/L) were within normal ranges. To confirm the diagnosis of AE for the two boys, direct sequencing analysis of SLC39A4 (ENST00000301305) was conducted on the family. The result showed that they were compound heterozygous for a novel missense mutation (c.926G > T) in exon 5 and a novel splicing mutation in the donor site of intron 5 (c.976 + 2 T > A) in SLC39A4. Among the two mutations, c.926G > T was originated from their mother, leading to a substitution of the 309th amino acid residue cysteine with phenylalanine (p.Cys309Phe), and c.976 + 2 T > A was inherited from their father, suggesting that it would alter splicing of the mRNA of SLC39A4 (Table 1,Fig. 3). On these bases, the patients were diagnosed with AE.

**Table 1:** The details of the genetic variants found in this case

Gene	the chromosomal position of the mutation	the mRNA accession	nucleotide changes	protein changes in HUGO gene nomenclature format	SIFT	PolyPhen	Mutation type
SLC39A4	8q24.3 exon 5	NM-130849	c.926G > T	p.Cys309Phe (p.C309F)	0.006	score:0.767;sensitivity: 0.85; specificity: 0.92	missense
SLC39A4	8q24.3 intron 5	NM-130849	c.976 + 2 T > A	–	–	–	a splice site mutation

“-” No information is displayed because the mutation site is located in the intron; SIFT: Score 0–0.05, predicted as damaging; PolyPhen: this mutation is predicted to be possibly damaging

**Fig. 4:**
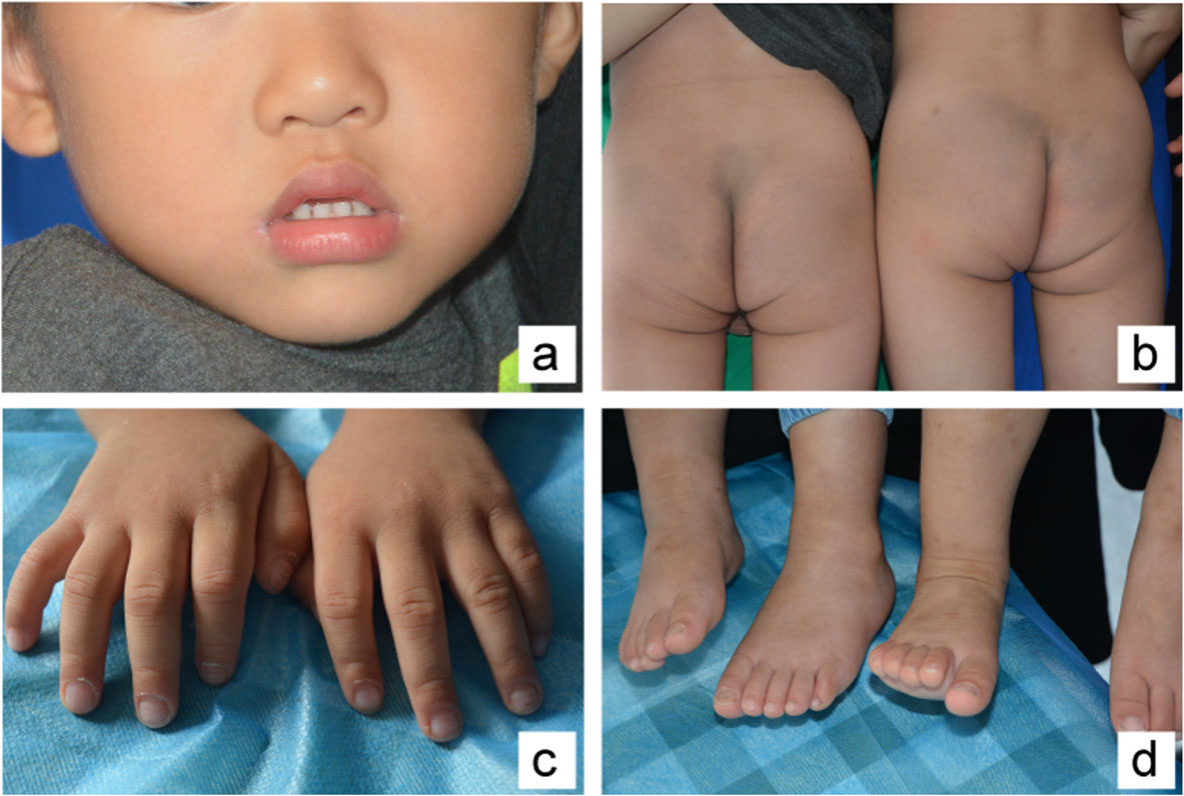
Skin performance after 1 year of treatment with 16 months of twins: **a** Face. **b** Perineum. **c** Hands. **d** Feet

The twins were treated with approximately 1.4 mg/kg of zinc per day. Topical zinc oxide oil and paste were applied to the lesions and ulcers. Vitamin E cream was applied to the body to keep the skin moist. After 3 months of continuous medication, the lesions were completely resolved. After supplementing zinc for 1 year, the disease never relapsed (Fig. 4).

## Discussion and conclusions

In the case, the boys had dermatitis at the ostium, accompanied by wrinkles, distal extremities and nail damage that appeared 5 months after weaning. And Sanger sequencing revealed that they were both compound heterozygous for c.926G > T and c.976 + 2 T > A in SLC39A4. And in silico analysis with the online software MutationTaster showed that both these two mutations were predicted to be disease causing. Then the final diagnosis of AE for the twins was made. After 2 weeks of zinc supplementation, the condition remarkably improved. On this basis, we diagnosed the twins with AE. Differential diagnoses to be considered were atopic dermatitis, Olmsted syndrome, congenital ichthyosis and biotinidase deficiency. After exclusion, the twins were orally supplemented with 1.4 mg/kg zinc per day. No sign of recurrence was observed.

The human SLC39A4 gene covering approximately 4.5 kb of chromosomal region 8q24.3 consists of 12 exons and 11 introns [9]. According to the statistical results in Table 2 (refer to the Additional file 1: Table S1 for details), the average age of onset of AE was 9.81 months. Almost all patients had perioral skin or mucosal damage. Perineum partial dermatitis occurred in 92.59% of patients, and two patients with exon 10 mutations were less prone to dermatitis in this area. Nail and other systemic impairments showed different clinical phenotypes due to variations in genetic mutation locations. The proportions of dermatitis in the wrinkles of the trunk, extremities, nail damage, alopecia, diarrhoea, irritability and serum zinc levels were 71.43, 85.71, 42.86, 39.29, 42.86, 25 and 67.86%, respectively.

**Table 2:** Review the clinical features of AE patients in 27 cases

patients	Age of onset (month)	Dermatitis site	Nail involvement	Alopecia	Diarrhea	Growth delay	Neuropsychiatric disorders	serum zinc levels	References
P1	7	1,2,3	0	0	0	0	0	0	[2]
P2	10	1,2,3	0	1	0	1	1	1	[10]
P3	6	1,2,3,4	0	0	0	0	0	1	[4]
P4	60	1,2,3,4	0	0	0	1	0	1	[4]
P5	2	1,2,3,4	0	0	1	0	0	1	[4]
P6	10	1,3,4	1	0	0	0	0	1	[5]
P7	10	1,3,4	1	0	0	0	0	1	[5]
P8	7	1,3,4	1	0	0	0	0	1	[5]
P9	12	1,2,3,4	1	1	0	0	0	1	[1]
P10	8	1,3,4	1	0	0	0	0	1	[1]
P11	5	1,2,3	0	0	0	0	0	1	[10]
P12	5	1,2,3	0	0	0	0	0	–	[10]
P13	8	1,4	1	0	1	0	0	0	[11]
P14	3	1,2,3,4	1	1	1	1	1	1	[12]
P15	12	1,2,4	0	1	1	0	0	1	[13]
P16	12	1,4	0	1	0	0	1	–	[14]
P17	12	1,2,3,4	1	1	1	1	1	–	[15]
P18	12	1,3,4	0	1	1	0	0	1	[16]
P19	5	1,2,3,4	0	0	1	1	0	1	[17]
P20	2	1,2,3,4	0	1	0	0	0	0	[2]
P21	15	1,2,3,4	0	0	1	1	1	1	[18]
P22	7	1,2,3,4	0	1	1	0	0	1	[19]
P23	1	1,2,3,4	0	0	1	0	0	1	[20]
P24	5	1,2,3,4	1	1	1	1	1	1	[8]
P25	5	1,2,3,4	1	1	1	1	1	1	[8]
P26	12	1,2,3,4	1	0	0	0	0	0	this report
P27	12	1,2,3,4	1	0	0	0	0	0	this report

Dermatitis site: Perioral = 1,Torso fold = 2,Limb end = 3,Perineum = 4; Other clinical manifestations: yes = 1;no = 0. Serum zinc levels: low = 1,normal = 0; “-” not available

The four twins we currently know of are from Asian countries, including the twins we have described herein. These patients are identical twins, mainly with skin erythema blistering as the main manifestation, serological examination and lighter skin performance. Zinc supplementation was applied at 1–5 mg / kg.d, and the condition generally improved after approximately 2 weeks. Statistics have shown that the common high-frequency mutations of AE are in exons 9, 3 and 5 (Table 3). Missense mutations account for 71.43% of the gene phenotype. Exon 9 mutations can occur in men and women and have an average onset age of 15.86 ± 9.21 months. The clinical manifestations are mainly skin and mucosal damage. In addition to the common perioral and perineal lesions, the clinical phenotype of the exon 9 mutation has the following characteristics. The extremities and the folds of the trunk are often involved. Damage and growth retardation are rare. Symptoms, such as mental irritability, are almost non-existent.

**Table 3:** Panel of SLC39A4 deleterious mutations noted in AE patients

	Exon 9	Exon 3	Exon 5
Number of patients	7	4	3
The gender ratio	17.15%	25.00%	1
gene mutation(Main)	missense	missense	missense
Age of onset (month)	15.86 + 9.21	6.25 + 1.71	10.33 + 1.18
Perioral(N)	1	1	1
Torso fold(N)	5	3	2
Limb end(N)	6	3	1
Perineum(N)	1	1	1
Nail involvement(N)	2	3	1
Alopecia(N)	3	1	0
Diarrhea(N)	3	3	0
Growth delay(N)	1	3	0
Neuropsychiatric disorders(N)	0	1	0
low serum zinc levels(N)	1	1	1
therapeutic dose(mg/kg.d)	2.64 + 1.03	4.25 + 1.71	1 + 0
course of treatment(day)	14.86 + 2.06	12.5 + 2.95	14 + 0

The gender ratio: the ratio of men to the total number of people; the ratio of the number of patients with positive clinical manifestations to the total number of patients with the same type of gene mutation; N: number of patients with this symptom

Exon 3 mutations can occur in males and females with an average incidence of 6.25 ± 1.71 months. In addition to the classic three clinical manifestations of AE, this type of mutation can have serious clinical symptoms, such as vertigo in the trunk folds and distal extremities. Mutations include pustules and clam shells, severe nail depression, hair loss, diarrhoea growth retardation and intermittent mental irritability. Almost all patients with this type of mutation have decreased plasma zinc levels. The age of onset of the exon 3 mutation is lower than the average, and the clinical symptoms are heavy at onset stage. Multiple systemic damages may occur, thus requiring the clinician to give a large zinc supplementation dose. Similar to an early treatment, the prognosis is enhanced.

The exon 5 mutation in this article presents generally mild clinical manifestations. The patients were all male, and the average age of onset was 10.33 ± 1.18 months. Only vaginal dermatitis, nail apex irregularities, alopecia, diarrhoea and delayed growth were observed, and no mental symptoms appeared. Plasma zinc and alkaline phosphatase levels were unspecific, and oral zinc supplementation has improved rapidly, and long-term maintenance of low-dose zinc supplementation has achieved satisfactory results. This vitamin allows the body to slowly supplement zinc for a long time, is conducive to stimulating the absorption and metabolism of zinc in children, and substantially improves the disease and permits clinical treatment without the fear of long-term side effects.

In conclusion, we identified an unreported compound heterozygous mutation in SLC39A4 was discovered in Chinese twins with AE. Summary analysis revealed variations in the phenotypes caused by distinct exon mutations in this gene and in the severity and prognosis of the disease. This work provides several suggestions for clinical diagnosis and genetic counseling of AE.

## Supplementary information

**Additional file 1: Table S1.** Details of clinical features of AE patients in 27 cases.

## Data Availability

All data generated or analysed during this study are included in this published article.

## Abbreviation

AE; OMIM 201100: Acrodermatitis enteropathica
